# Human Activities on the Deep Seafloor in the North East Atlantic: An Assessment of Spatial Extent

**DOI:** 10.1371/journal.pone.0012730

**Published:** 2010-09-13

**Authors:** Angela R. Benn, Philip P. Weaver, David S. M. Billet, Sybille van den Hove, Andrew P. Murdock, Gemma B. Doneghan, Tim Le Bas

**Affiliations:** 1 Ocean Biogeochemistry and Ecosystems Group, National Oceanography Centre Southampton, Southampton, United Kingdom; 2 Geology and Geophysics Group, National Oceanography Centre Southampton, Southampton, United Kingdom; 3 Median, Valldoreix, Spain; 4 Institut de Ciència i Tecnologia Ambientals, Universtitat Autònoma de Barcelona, Barcelona, Spain; 5 GeoData Institute, University of Southampton, Southampton, United Kingdom; California Academy of Sciences, United States of America

## Abstract

**Background:**

Environmental impacts of human activities on the deep seafloor are of increasing concern. While activities within waters shallower than 200 m have been the focus of previous assessments of anthropogenic impacts, no study has quantified the extent of individual activities or determined the relative severity of each type of impact in the deep sea.

**Methodology:**

The OSPAR maritime area of the North East Atlantic was chosen for the study because it is considered to be one of the most heavily impacted by human activities. In addition, it was assumed data would be accessible and comprehensive. Using the available data we map and estimate the spatial extent of five major human activities in the North East Atlantic that impact the deep seafloor: submarine communication cables, marine scientific research, oil and gas industry, bottom trawling and the historical dumping of radioactive waste, munitions and chemical weapons. It was not possible to map military activities. The extent of each activity has been quantified for a single year, 2005.

**Principal Findings:**

Human activities on the deep seafloor of the OSPAR area of the North Atlantic are significant but their footprints vary. Some activities have an immediate impact after which seafloor communities could re-establish, while others can continue to make an impact for many years and the impact could extend far beyond the physical disturbance. The spatial extent of waste disposal, telecommunication cables, the hydrocarbon industry and marine research activities is relatively small. The extent of bottom trawling is very significant and, even on the lowest possible estimates, is an order of magnitude greater than the total extent of all the other activities.

**Conclusions/Significance:**

To meet future ecosystem-based management and governance objectives for the deep sea significant improvements are required in data collection and availability as well as a greater awareness of the relative impact of each human activity.

## Introduction

Environmentally sustainable governance and management requires the availability of reliable and comprehensive information on the natural environment as well as information on the social, economic, legal and political systems. However, even though the deep seafloor covers approximately 60% of Earth's surface [1] only about 0.0001% of it has been the focus of biological scientific investigation [2]. Whilst remoteness and inaccessibility restrict research, they have not protected these depths from human impacts. Increasing demand for living and non-living resources and diminishing or exhausted reserves on land and in shallow water are pushing human activities ever deeper into the world's oceans. At the same time advances in technology now allow access to resources of economic value that were previously inaccessible. This has resulted in an increasing number of direct and indirect anthropogenic pressures on deep-sea ecosystems [1]–[6].

**Figure 1:**
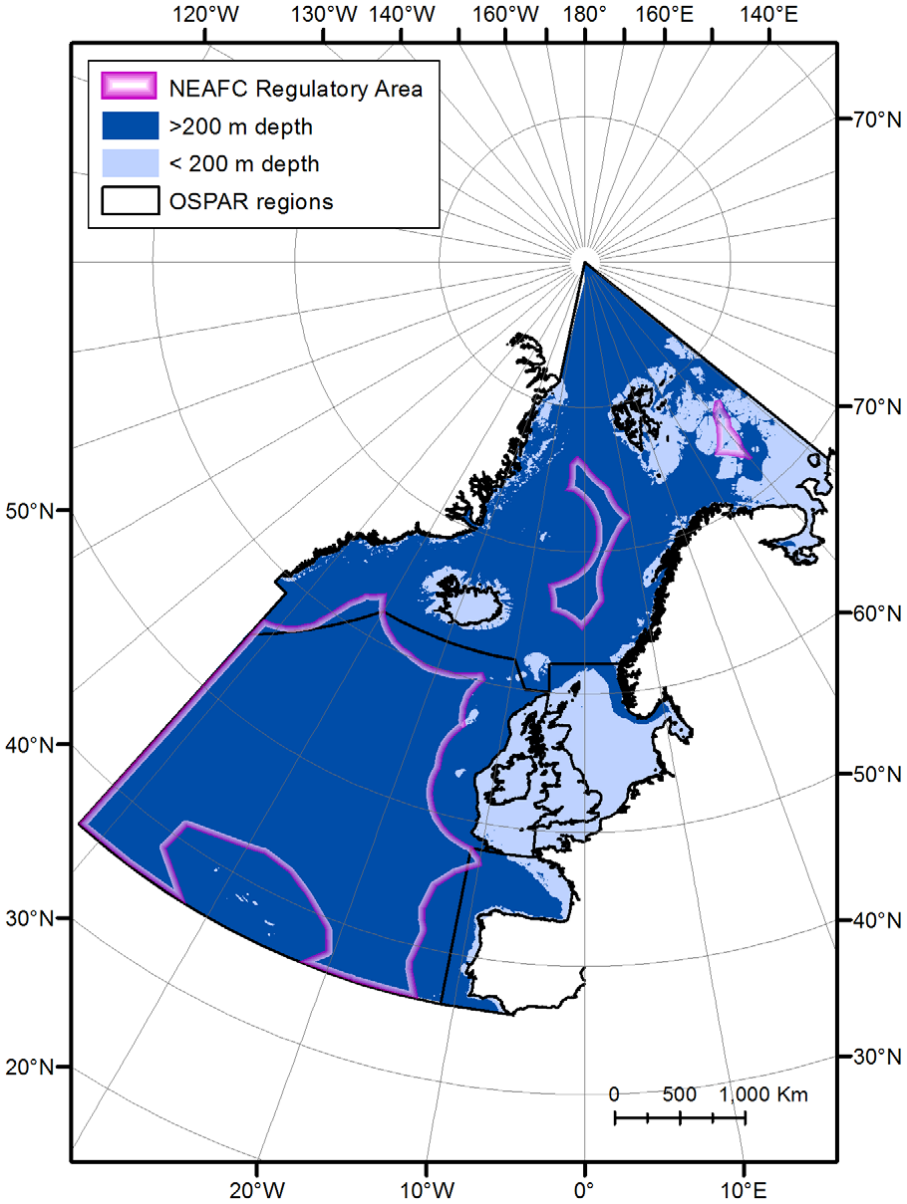
North East Atlantic Fisheries Commission Regulatory Area and OSPAR Maritime Area. OSPAR Regions I: Arctic Waters, II: Greater North Sea, III: Celtic Seas, IV: Bay of Biscay and Iberian Coast, V: Wider Atlantic.

Governance and management of the deep sea is of increasing international concern. The United Nations, the Regional Seas conventions and organisations, including the European Union, are developing marine environment policies as well as monitoring and reporting procedures. Rules and codes of conduct are being established to regulate activities impacting on the deep ocean. For example, the OSPAR Commission has recognised the scientific case for establishing Marine Protected Areas in areas beyond national jurisdiction in the deep North East Atlantic e.g. [7]. It has developed a code of conduct for Responsible Marine Research in the Deep Seas and High Seas of the OSPAR Maritime Area [8] (Figure 1). The North East Atlantic Fisheries Commission (NEAFC) (Figure 1) has adopted procedures and rules for existing and new bottom-fishing areas aimed at the protection of vulnerable marine habitats [9]–[12]. NEAFC and the OSPAR Commission have initiated the first efforts towards multi-sectoral management in the High Seas in the North East Atlantic. Under a new memorandum of understanding, adopted by the two organisations in 2008, an attempt is being made to combine fisheries and conservation management [7].

The requirement for environmental and socio-economic data is recognised in many political forums. The 1995 United Nations (UN) Fish Stocks Agreement calls for the sharing of “complete and accurate data concerning fishing activities” [13]. The Convention on Biological Diversity [14] promotes the ecosystem approach as its primary framework for action. The ecosystem approach is a strategy for the integrated management of land, water and living resources that promotes conservation and sustainable use in an equitable way, recognizing that humans and their activities are integral to ecosystems. At the European level, the Marine Strategy Framework Directive (MSFD) [15] and the OSPAR Biological Diversity and Ecosystems Strategy [16] both require assessments of human activities within the marine environment, some of which will be in the deep sea and beyond national jurisdictions. To fulfill these assessments and to implement the ecosystem approach, comprehensive and consistent information on human activities is necessary.

Data on human activities are collected and held i) by public institutions and private companies to fulfill regulatory requirements, ii) for commercial and operational purposes and iii) or for scientific research. In addition, the European Union Directive on Public Access to Environmental Information [17] defines environmental information to include “measures (including administrative measures), such as policies, legislation, plans, programmes, environmental agreements, and activities affecting or likely to affect the elements and factors …”. These include “… water, soil, land, landscape and natural sites, … marine areas, biological diversity and its components, … and the interaction among these elements”.

This study assesses, for the first time, the relative spatial extent of major human activities in the deep North East Atlantic, within and beyond Exclusive Economic Zones (EEZs) in the OSPAR maritime area of the North Atlantic (Figure 1), of which 8,517,010 km^2^ is deeper than 200 m, during the single year, 2005. The marine ecosystems here are some of the most heavily impacted by human activities [18]. The availability and suitability of data relating to these activities are assessed and the spatial extent of the direct physical impact on the seafloor is quantified. However, the extent of collateral physical impacts, for example smothering caused by sediment plumes and chemical effects on the benthos, for example those related to oil industry cuttings piles, are not assessed. In addition, we do not estimate the wider chemical and biological impacts caused by pollution. In the current study, “human activities”, identified by reference to literature [1]–[5], are defined as intentional human activities occurring directly on the sea floor as well as structures and artefacts present on the seafloor resulting from past activities. Previous studies in shallower waters have examined much smaller areas in detail [19], [20] , or have looked at single activity impacts [21], whilst some studies such as Halpern et al. [18] have taken a broad global view.

## Methods

Data for activities were requested from sources listed in Tables 1, 2 and 3. They were rarely in a format immediately suitable for assessing the spatial extent of each activity. Typically, data were provided as text files or MS Excel sheets with XY point locations of features; for example marine scientific research sample sites or radioactive dumpsites. In the case of vessel tracks or pipelines data were either strings of coordinate points (in text files or MS Excel) or actual GIS datasets (polyline features). As such, these have no areal definition but merely describe the route a vessel took based on its GPS track or location of a point on the seabed.

**Table 1:** Data sources.

Source	Contact information
**Marine Scientific Research**	
Report of Observations/Samples collected by Oceanographic Programmes (ROSCOP) Cruise Summary Reports	http://www.ices.dk/Ocean/roscop/index.asp
British Oceanographic Data Centre (BODC)	http://www.bodc.ac.uk
Hotspot Ecosystem Research on the Margins of European Seas (HERMES)	http://www.eu-hermes.net/members/cruises.html
Intergovernmental Oceanographic Commission of UNESCO, International Oceanographic Data and Information Exchange	http://www.oceandataportal.org
National Marine Facilities, National Oceanography Centre, Southampton	http://www.noc.soton.ac.uk/nmf
Ocean Information Centre, Research Ship Schedules and Information	http://www.researchvessels.org
Pangaea Publishing Network for Geoscientific & Environmental Data	http://www.pangaea.de
Various individual scientific institutions	
**Submarine Cables**	
Kingfisher Information Service – Cable Awareness	www.kisca.org.uk/charts.htm#option4
France Telecom SigCables	www.sigcables.com/cgi-bin/index.pl
**Waste disposal: Radioactive Waste**	
NEA.1985. Review of the Continued Suitability of the Dumping Site for Radioactive Waste in the North-East Atlantic. Nuclear Energy Agency, Organisation for Economic Cooperation and Development, Paris. 448pp.	
**Waste Disposal: Munitions and chemical weapons**	
OSPAR. 2005. (Revised). Overview of Past Dumping at Sea of Chemical Weapons and Munitions in the OSPAR Maritime Area. Biodiversity Series. OSPAR, London. 13 pp.	http://www.ospar.org/documents%5Cdbase%5Cpublications%5Cp00222_2005%20Revised%20Dumping%20at%20Sea%20of%20chemical%20weapons.pdf
**Oil and Gas Industry**	
UK Digital Energy Atlas and Library	http://www.ukdeal.co.uk
Norwegian Petroleum Directorate	http://www.npd.no/en/

Sources from which data were acquired.

**Table 2:** Military activities.

Source	Contact Information
NATO	mailbox.natodoc@hq.nato.intscience@hq.nato.int
French Ministry of Defence	http://www.defense.gouv.fr/formulaire_de_contact
Norwegian Ministry of Defence	postmottak@fd.dep.no
Portuguese Ministry of Defence	gcrp@defesa.pt
Spanish Ministry of Defence	comunicacion@fn.mde.es
Irish Defence Forces (Freedom of Information request)	foi@defenceforces.ie
UK Ministry of Defence (Freedom of Information request)	http://www.mod.uk/DefenceInternet/ContactUs/FreedomOfInformationInformationRequest.htm
Government of Greenland	info@gh.gl
Government of Iceland	external@utn.stjr.is

Sources to which requests for information on military activities during 2005 in the North East Atlantic were addressed.

**Table 3:** Sources to which requests for VMS data were addressed.

State	Source	Contact
†Denmark	Fiskeridirektoratet	sat@fd.dk
†France	Cross Atlantique	Csp-France.CROSS-Etel@developpement-durable.gouv.fr
Greenland	Fisheries Authority	APNA@gh.gl
Iceland	Ministry of Fisheries and Agriculture	postur@slr.stjr.is
†Ireland	Fisheries Monitoring Centre	nscstaff@eircom.net
Norway	Ministry of Fisheries and Coastal Affairs	postmottak@fkd.dep.no
†Portugal	Direcção Geral das Pescas e Aquicultura, Departamento de Inspecção das Pescas	ccc@ip.dgpa.min-agricultura.pt
†Spain	Secretaría General de Pesca Maritíma	csp@mapya.es
†UK	Marine Fisheries Agency Data and Communications	sat.ops@mfa.gsi.gov.uk

† EC Fishing Monitoring Centres Contact List: http://ec.europa.eu/fisheries/cfp/control/fmc_contact_list_en.pdf.

To define a realistic areal footprint for features, the data were processed in ArcGIS v. 9.3 (Environmental Systems Research Institute) for processing. This industry standard GIS package has tools for ‘buffering’ spatial features by a specified width (or range of widths). The output of this processing is a polygon shape which is a proxy for the actual spatial location and extent of the features on the seabed (the footprint). The tools operate on point or polyline features and can be used in a variety of coordinate systems.

ArcGIS's implementation of the *North Pole Lambert Azimuthal Equal Area Conic projection* was chosen as appropriate for use within the OSPAR regional extent and is designed to minimise area distortions.

Some of the datasets contained the necessary information to create the areal footprint, for example, known diameters of oil industry pipelines. Where this information was unavailable, values were sought from owners of the assets, industry experts or from published literature values.

Depth zones were identified by reference to the GEBCO dataset (General Bathymetric Chart of the Oceans) [22]. GEBCO is a world bathymetry dataset on a 1 arc minute grid and is the most extensive freely available bathymetric dataset.

**Figure 2:**
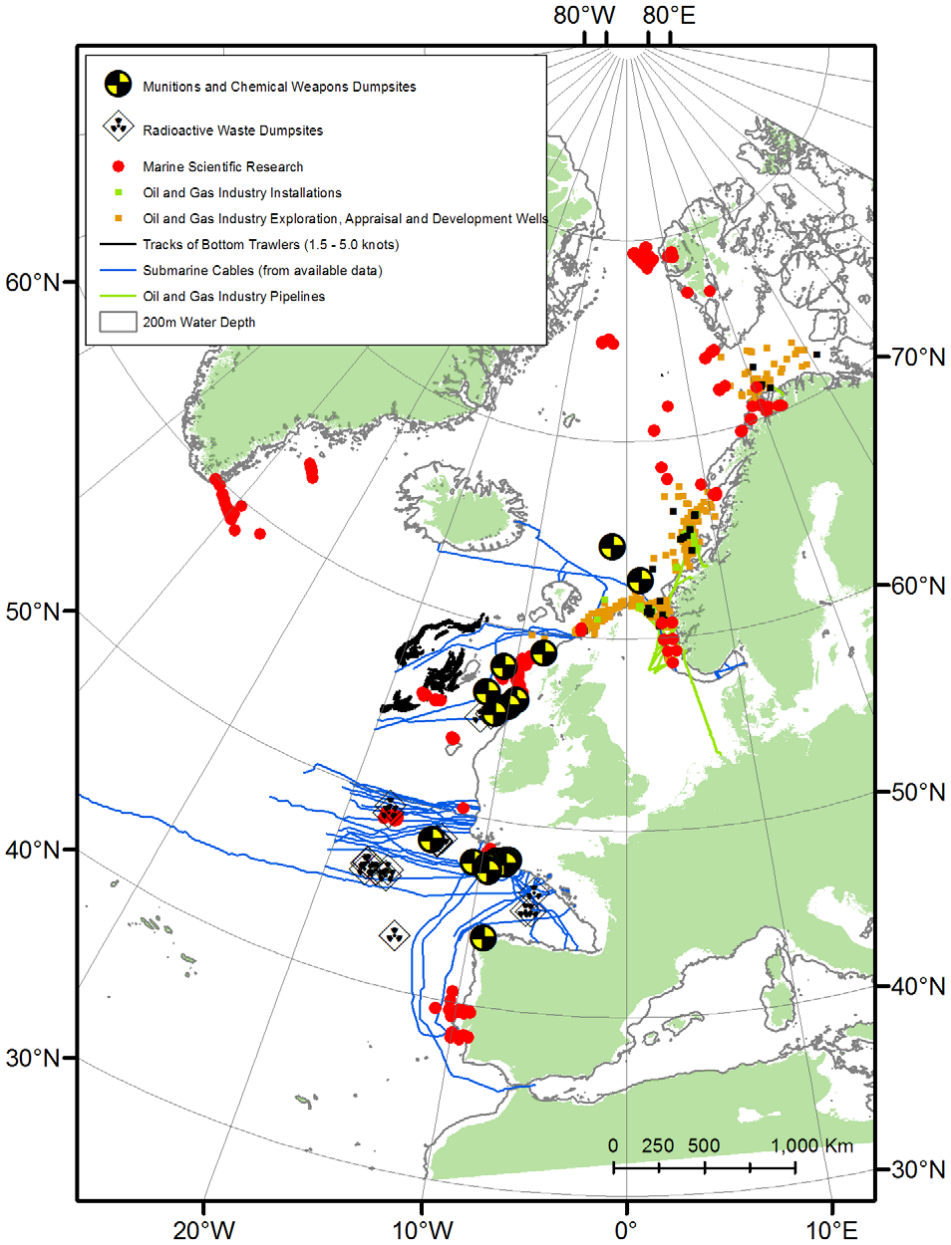
Human activities on the seafloor, including structures and artefacts present on the seafloor resulting from past activities, within the OSPAR Maritime Area, >200 m water depth, during 2005.

Buffer polygons were created for each feature and the area values (automatically created by the GIS) were extracted and totalled to estimate the spatial extent of each activity (Table 4). A confidence rating relating to the quality of data was applied, based on the method described by Eastwood et al. [19]. A score of 1 denotes an estimated location and extent; 2 denotes a known location but estimated extent and 3, a known location and extent. Figure 2 shows the geographical distribution of activities. Where the data used to calculate the estimates did not represent the total extent of an activity in the OSPAR deep water area, (marine research, submarine cables and bottom trawling) a further estimate, extrapolated to represent the total of each activity, was calculated (Table 5).

**Table 4:** Spatial extent and confidence rating of activities.

Activity	Estimated spatial extent	Confidence rating†
(>200m water depth)	(km^2^)	
**Scientific research:** (estimated 45% of all cruises impacting on seafloor during 2005)		
Non-fisheries research cruises	4	2–3
Fisheries research cruises	22	2–3
**Submarine communications cables:** (estimated 41% of all submarine cables)		
No burial: between 200–1500 m wd, 50 mm diameter cable ; >1500 m wd, 20 mm diameter cable	2	1–2
No burial: between 200–>1500 m wd, 50 mm** diameter cable	4	1–2
Cable burial: between 200–1500 m wd with 2 m wide disturbance strip*; no burial >1500 m wd, 20 mm diameter cable	15	1–2
Cable burial: between 200–1500 m wd with 8 m wide disturbance strip*; no burial >1500 m wd, 20 mm diameter cable	61	1–2
**Waste disposal:**		
Radioactive waste	0.2	2
Munitions and chemical weapons	1.4	1
**Military**	No data made available	
**Oil and gas:**		
Pipelines	4.0	3
1 ^,^ 2Structures: platforms, templates and wellheads	0.2	2
2Structures with associated cuttings piles (∼83 m radius^3^)	3	2
2Wells drilled during 2005 with associated cuttings piles (∼83 m radius^3^)	1	2
2Wells drilled between 1960 and December 2005 and associated cuttings piles (∼83 m radius^3^)	15	2
Total pipelines, structures, wells and cuttings piles	23.2	2–3
**Bottom trawling: (2005, Hatton and Rockall area)**		
**- Speed range 2.0–3.0 knots, gear width 22 m:**		1–2
Tracks not merged	741	
Tracks merged	548	
**- Speed range 1.5–5.0 knots, gear width 125 m:**		1–2
Tracks not merged	37,160	
Tracks merged	13,920	

Estimates of the spatial extent of six major human activities occurring directly on the sea floor, including structures and artefacts present on the seafloor resulting from past activities, within the OSPAR maritime area of the North East Atlantic in waters >200 m during 2005. Estimates for bottom trawling and marine scientific research are based on 2005 data only.

wd: water depth;

† Confidence ratings indicate whether the spatial extent of each activity is based on data or estimates of location and extent (Eastwood et al., 2007) [19]: 1, estimated location and estimated extent; 2 known location, estimated extent; 3, known location and extent.

*Carter et al., 2009 [23].

1 Information from NPD and Statoil datasets and Eastwood et al., 2007 [19].

2 Overlapping boundaries merged.

3 SERPENT Project, unpublished data.

**Table 5:** Comparison of extrapolated spatial extent of human activities in the OSPAR area in 2005.

Activity	Estimated spatial extent	Extrapolated to 100% of activity
(>200m water depth)	(km^2^)	(km^2^)
**Scientific research:** 45% of cruises with activities on the seafloor reported to ROSCOP during 2005		
Non-fisheries research cruises	4	9
Fisheries research cruises	22	49
**Submarine communications cables:** Estimate based on 41% of cables		
No burial: between 200–1500 m wd, 50 mm diameter cable ; >1500 m wd, 20 mm diameter cable	2	5
No burial: between 200–>1500 m wd, 50 mm** diameter cable	4	10
Cable burial: between 200–1500 m wd with 2 m wide disturbance strip*; no burial >1500 m wd, 20 mm diameter cable	15	Extrapolation inappropriate – see text.
Cable burial: between 200–1500 m wd with 8 m wide disturbance strip*; no burial >1500 m wd, 20 mm diameter cable	61	Extrapolation inappropriate – see text.
**Waste disposal:** Includes all recorded data		
Radioactive waste	0.2	0.2
Munitions and chemical weapons	1.4	1.4
**Military**	No data made available	No data made available
**Oil and gas:** Includes all recorded data and extrapolations		
Pipelines	4	4
1 ^,^ 2Structures: platforms, templates and wellheads	0.2	0.2
2Structures and associated cuttings piles (∼83 m radius^3^)	3	3
2Wells drilled during 2005 and associated cuttings piles (∼83 m radius^3^)	1	1
2Wells drilled between 1960 and December 2005 and associated cuttings piles (∼83 m radius^3^)	15	15
Total pipelines, structures, wells and cuttings piles	23.2	23.2
**Bottom trawling in Hatton and Rockall during 2005** estimated as ∼50% of all deep sea bottom trawling area in the OSPAR area		
**- Speed range 2.0–3.0 knots, gear width 22 m:**		
Tracks not merged	741	1,482
Tracks merged	548	1,096
**- Speed range 1.5–5.0 knots, gear width 125 m:**		
Tracks not merged	37,160	74,320
Tracks merged	13,920	27,840

Estimates and extrapolations of the spatial extent of six major human activities occurring directly on the sea floor, including structures and artefacts present on the seafloor resulting from past activities, within the OSPAR maritime area of the North East Atlantic in waters >200 m during 2005. Estimates for bottom trawling and marine scientific research are based on 2005 data only.

wd: water depth;

*Carter et al., 2009] [23].

1 Information from NPD and Statoil datasets and Eastwood et al., 2007 [19].

2 Boundaries merged and dissolved.

3 SERPENT Project, unpublished data.

The datasets were drawn from a variety of sources. They were collected for a variety of purposes. Some data were only indicative. Some were derived from GPS tracking. Others were surveyed precisely. Therefore, positional accuracies varied. This is a broad scale strategic study and while it is important to obtain as accurate information as possible, the study is considering the *relative* spatial extent of these activities in the context of the OSPAR region, and small errors are not likely to be significant to the final values. The study quantifies the physical footprint but does not quantify how significant (detrimental or beneficial) these impacts might be on the surrounding ecosystems. This study does not tackle contamination that may be spread away from the specific impact e.g. leakage of radioactivity.

### Marine Scientific Research

Marine scientific research is carried out by academic institutions or fisheries research laboratories. Research by academic institutions involves a range of equipment on the seafloor to sample the marine environment including moorings, grabs, corers, dredges and trawls. Much of this equipment has only a single impact of a few square meters. While fisheries research also involves the deployment of sampling equipment, such as grabs and moorings, it involves a higher proportion of bottom impact trawling.

Data were obtained from the seven online sources listed in Table 1 and individual scientists. Twenty four cruises, which took place in water deeper than 200 m and carried out activities on the seafloor, were identified from cruise reports and station lists. A further 29 cruises which may have impacted on the seafloor in water deeper than 200 m were accessed on the ROSCOP website but searches in PANGAEA, BODC and European project databases (e.g. HERMES) did not locate station lists or cruise reports. Cruises for which data were available represent approximately 45% of the total number of cruises identified during 2005 which may have impacted on the seafloor within the OSPAR area listed on the ROSCOP cruise summary. Where cruise reports and station lists were available activities on the seafloor were mapped. According to the footprint size of each piece of equipment buffers were applied to estimate the spatial extent on the seafloor. Where the footprint area of each activity was not included in the cruise report (size of equipment deployed, length and width of trawl) it was estimated based on published literature and advice from individual institutions.

### Submarine Communication Cables

Greater than 95% of international communications are routed via submarine fibre-optic cables. In areas where cables are vulnerable to damage from fishing or anchoring (200–1,500 m water depth) they often have one or more layers of armour and can be up to 50 mm in diameter. In waters deeper than 1,500 m, currently beyond the reach of fishing, cables are non-armoured and are between 17 mm and 20 mm in diameter [23]. An alternative protective measure is the burial of cables in water depths shallower than 1,500 m [23]. During the burial operation a plough opens a furrow in the seafloor into which the cable is laid and the sediment replaced. Skids supporting the plough can leave a footprint on the seabed, particularly in zones of soft sediment, potentially increasing sediment compaction and leading to the disturbance of the marine fauna. The overall width of the disturbance strip produced by the plough-share and skids in direct contact with the seabed ranges from 2 to 8 m width [23]. The spatial extent calculated here represents the width of either the unburied cables on the seafloor or, for buried cables, the footprint of the plough based on the minimum and maximum width of disturbance strips (2 m and 8 m) [23], although it is unlikely that the disturbance strip is 8 m everywhere.

Geospatial data for submarine cables were obtained from the two sources listed in Table 1. Kingfisher Information Service – Cable Awareness data were available in Microsoft Excel format to an accuracy of 10 m and France Telecom's SigCables, available as ESRI shape files. These websites, for users of the seabed and, in particular, for skippers of fishing vessels, give cable locations to approximately 25°W, beyond which the water is too deep for the cables to be in danger. As no data were available beyond ∼25°W, the cable lines were extrapolated from the final data point provided for each cable to a landfall in the United States or Canada, identified from ICPC, 2008 [24]. The distance to the western boundary of the OSPAR maritime area, 42°W was calculated. Forty five cables were identified with an approximate total length of 75,055 km, which included all of the current in-service systems as at 2005. However, this does not take into account all systems dating back to the start of telegraphic communications. The total approximate length of all cables (including coaxial, fibre optic and telegraph cables but not including military) on the seafloor within the OSPAR area during 2010 is estimated at 184,200 km (Steve Bennett, Global Marine Systems Limited, personal communication). This is the nearest total value obtainable by the study. The spatial extent of cables calculated within this study is estimated to represent approximately 41% of the total area of cables.

Neither dataset reported whether the cables were buried, armoured or non-armoured. Therefore, 4 scenarios have been considered based on the following assumptions:

1. No cable burial at any water depth. Cable diameter 50 mm in water depths 200 m–1,500 m and 20 mm diameter in water depths greater than 1,500 m.
2. No cable burial at any water depth. Cable diameter of 50 mm at all water depths (the maximum diameter of modern, double armoured fibre optic cables [23]).
3. In water depths between 200 m–1,500 m cables buried by a plough with an overall disturbance footprint of 2 m width – the minimum width reported [23]. In water depths greater than 1,500 non-buried cable, 20 mm diameter.
4. In waters depths between 200 m–1,500 m cables buried by a plough with an overall disturbance footprint of 8 m width - the maximum width reported [23]. In water depths greater than 1,500 non-buried cable, 20 mm diameter.

The data were input into ArcGIS. Cables whose entire length was in water <200 m depth were removed from the dataset. The lines depicting the cables were segmented to account for the different depth zones (200–1,500 m and >1,500 m). The relevant depth zones were extracted from the GEBCO dataset. The linear features were intersected with the depth zones, splitting the line at the boundaries of the zones and the sections were attributed with the required width values (50 mm, 20 mm, 2 m and 8 m). This allowed variable buffers to be created for different sections of each line. The depth contours were simplified in areas of complex geomorphology to avoid adding spurious detail to the calculations. Cables crossing areas of Mid-Atlantic Ridge at depths <1,500 m were assumed to be 20 mm diameter as there is no cable burial or armouring in this area.

### Waste Disposal

This study focused on chemical and conventional munitions and low level radioactive waste dumped prior to the 1996 London Protocol [25]. This protocol came into force on 24 March 2006 and recognised seven categories of waste; i) dredged material; ii) sewage sludge; iii) fish waste (or material resulting from industrial fish processing operations); iv) vessels and platforms or other man-made structures at sea; v) inert, inorganic geological material; vi) organic material of natural origin. The seventh category includes “bulky items primarily comprising iron, steel, concrete and similar unharmful materials for which the concern is physical impact and limited to those circumstances, where such wastes are generated at locations, such as small islands with isolated communities, having no practicable access to disposal options other than dumping” [25].

#### Radioactive waste

Between 1949 and1982 radioactive waste was dumped routinely at sites in the North East Atlantic. It included i)‘low level’ wastes from nuclear power plant operations; ii) other nuclear fuel cycle operations, including fuel fabrication and reprocessing; iii) radionuclide use in medicine, research and industry and iv) decontamination and dismantling of redundant plant and equipment [26].

In 1983 increasing concern over the continued sea disposal of radioactive waste led the Contracting Parties to the London Convention [27] to adopt a voluntary moratorium on the sea dumping of all types of radioactive waste. Amendments to the Convention, adopted in 1993 , which came into force on 20 February 1994, eventually banned sea dumping of all types of radioactive waste [25]. Twenty five years from this date, contracting parties are required to complete a scientific study relating to all radioactive wastes and other radioactive matter other than high level wastes, followed by further studies at 25 year intervals [27].

Information relating to dumping sites for radioactive waste was obtained from a single source [26], (Table 1). An estimate of the total area designated for dumping of radioactive waste was 26,323 km^2^, based on the aggregated areas with overlapping boundaries dissolved for each of the four designated sites (Table 6). However, this does not represent the area of seafloor covered by drums of waste so a second estimate of the extent of this activity was based on the tonnage and estimated number of drums (Table 6). Thiel [5] estimates that, in total, between 1949 and 1982, 222,732 drums containing 114,726 tonnes (t) of radioactive waste were dumped at sites in the deep North East Atlantic. This is a mean of ∼0.5 t of waste per drum. Of the 42 dumping events listed in [26], 24 events totalling 112,793 t (Table 6) of waste were deposited in the OSPAR area in waters deeper than 200 m. A second estimate was calculated based on a mean of 0.5 t of waste per drum. It was estimated that there were 225,586 drums within the OSPAR area in waters deeper than 200 m with an approximate area of 1 m^2^ per drum [26].

**Table 6:** Radioactive waste dumpsites in water deeper than 200 m in the OSPAR region of the North East Atlantic between 1949 and 1984.

Longitude	Latitude	Year	Tonnes	Country of Origin	Description of Dumpsite
−16.75	46.00	1977	5 605	NL-CH-UK	a rectangle 45.8333 to 46.1666 and −16.00 to −17.50
		1978	8 046	B-NL-CH-UK	
		1979	5 416	B-NL-CH-UK	
		1980	8 391	B-NL-CH-UK	
		1981	9 434	B-NL-CH-UK	
		1982	11 693	B-NL-CH-UK	
−17.42	46.25	1971	3 968	B-NL-CH-UK	a circle of radius 35 nautical miles centred on 46.25, −17.41666
		1972	4 131	B-NL-CH-UK	
		1973	4 350	B-NL-UK	
		1974	2 265	NL-CH-UK	
		1975	4 454	B-NL-CH-UK	
		1976	6 772	B-NL-CH-UK	
−13.25	48.25	1965	1 760	UK	not described
		1966	1 044	UK	
−13.27	48.33	1970	1 674	UK	not described
		1968	3 164	UK	
−13.00	48.50	1949	9	UK	not described
−11.33	55.43	1951	33	UK	not described
−12.17	55.13	1953	57	UK	not described
−6.17	46.45	1962	253	UK	not described
−6.27	45.45	1963	5 809	B-UK	not described
−6.60	45.45	1964	4 392	UK	not described
−14.50	42.83	1967	10 895	B-F-D-NL-UK	a square of side 50 km centred on 42.83333, −14.5
−17.08	49.08	1969	9 178	B-F-I-NL-S-CH-UK	a square of side 50 nautical miles centred on 48.5, −17.08333
		**Total**	**112 793**		

Location of dumping area, quantities and sources of radioactive waste (based on NEA, 1985) [26].

B = Belgium; CH = Switzerland; D = Germany; F = France; I = Italy; NL = Netherlands; S = Sweden; UK = United Kingdom.

#### Munitions and chemical weapons

The locations of dumpsites for conventional and chemical munitions were identified by reference to [28] (Table 1). Of the 148 dumpsites recorded, 24 are in waters deeper than 200 m (Table 7). While the locations of dumpsites were reported, there was no indication of the area of each. However, twelve sites are described as a “scuttled ship”. Based upon this information a nominal square 100 m×100 m was assigned for each site.

**Table 7:** Conventional and chemical munitions dumpsites in waters >200 m in the OSPAR region (OSPAR, 2005) [28].

Site number	Longitude	Latitude	Type of munitions	Details
42	−13.66	48.33	Conventional	Only remaining UK dumpsite by 1993
43	−9.02	43.73	Conventional	
45	1.46	62.97	Chemical	4,500 tons scuttled vessels
46	−7.67	59	Chemical	
49	−11	58	Chemical	
51	−12.08	56.52	Chemical	
52	−12	56.5	Chemical	
53	−9.45	56.37	Chemical	
54	−10	56	Chemical	
55	−11	55.5	Chemical	
56	−9.37	48.67	Chemical	Scuttled ship, Dora Oldendorf - February 1947.
57	−8.15	48.05	Chemical	Scuttled ship, Empire Nutfield - September 1946.
58	−8.35	48	Chemical	Scuttled ship, Lanark - November 1946.
59	−8.56	47.95	Chemical	Scuttled ship, Empire Peacock - August 1946.
60	−8.97	47.92	Chemical	Scuttled ship, Harm Freitzen - March 1948.
61	−8.26	47.92	Chemical	Scuttled ship, Empire Lark - July 1947.
62	−8.35	47.9	Chemical	Scuttled ship, Kindersley - October 1946.
63	−8.85	47.87	Chemical	Scuttled ship, Empire Connyngham - June 1949.
64	−8.31	47.79	Chemical	Scuttled ship, Thorpe Bay - September 1947.
65	−10.5	47.63	Chemical	CW (Approx 70 Tonnes) encased in concrete. Dumped in 1980.
66	−9.52	47.6	Chemical	Scuttled ship, Margo - November 1947.
67	−9.4	47.38	Chemical	Scuttled ship, Miervaldis - September 1948.
68	−9.4	47.28	Chemical	Scuttled ship, Empire Success - August 1948.
70	−1.6	64.7	Chem. - Tabun	462 shells recovered in Wolgast Harbour dumped, set in concrete.

### Military Activities

It was not possible to estimate the spatial extent of this activity. Requests for information relating to military activities on the seafloor during 2005 were made to sources listed in Table 2. Only the Irish Defence Forces responded, reporting no activities on the seafloor deeper than 200 m during 2005. The UK Ministry of Defence redirected the request to the UK Hydrographic Office for locations of practice and exercise areas, but these provided no specific details of activities. The request to NATO was directed to the NATO Science Department which was unable to help.

### Oil and Gas Industry

Geospatial data for oil and gas industry subsurface installations, pipelines and exploration and development wells were obtained from the UK Digital Energy & Atlas Library (UKDEAL) [29] and the Norwegian Petroleum Directorate (NPD) [30] (Table 1).

The locations of pipelines were reported in the UK and Norwegian datasets but the diameter was recorded only in the UKDEAL data. Diameters for Norwegian pipelines were extracted individually from NPD Facts [31]. These data were imported into ArcGIS. Sections of pipeline in waters 200 m or deeper were identified and buffered to represent their respective diameters.

Neither the UKDEAL nor NPD datasets contained dimensions of other types of installations. Eastwood et al. [19] proposed two categories of installation, ‘platform’ and ‘well’ and assigned nominal areas of ∼180 m^2^ and a diameter of 50 m respectively. The UKDEAL datasets listed one platform and eleven wellheads in waters deeper than 200 m. Circular buffers of 180 m^2^ and 50 m diameter were applied to estimate the spatial extent of these features.

Most Norwegian deep water installations are floating platforms with wells drilled through templates on the seafloor. The original downloaded NPD dataset did not include the type of installation but, on request, a dataset was provided which included date installed and type of installation. In waters deeper than 200 m three platforms sited on the seafloor and 230 templates were listed. Four legs sit on the seabed supporting the template which typically covers 416 m^2^ of seafloor (Tore Indreiten, Statoil, personal communication). A square buffer of 416 m^2^ was applied to estimate the spatial extent of these installations and circular buffers of 180 m^2^ were applied to estimate the spatial extent of platforms.

In addition to structures on the seafloor, drill cuttings piles are a part of the footprint of oil and gas operations. A variety of oil-based, synthetic and water-based drilling fluids have been used, each with different technical and environmental properties [32]. Typically, cuttings piles are a mixture of man-made and natural substances containing higher concentrations of metals and hydrocarbons than background sediments. They consist of fragments of rock, mixed with drilling muds [33]. Discharge to the seafloor of oil-based drilling muds and associated cuttings ceased in 1993 and 1996 in Norway and the UK respectively. While water based drilling fluids and cuttings can, with permission, be discharged, used oil-based drilling fluids and cuttings are now either transported to land for processing or injected into the seafloor [34]. Recent photographic surveys carried out by the SERPENT Project (www.serpentproject.com) at exploration drilling sites in the Faroe-Shetland Channel and the Norwegian Sea indicate a mean area of 21,744 m^2^ is covered by drill cuttings in the deep sea (SERPENT Project, unpublished data). To estimate the spatial extent of oil and gas industry activities, including the presence of cuttings piles, a circular buffer of 21,744 m^2^ (radius of ∼83 m) was applied to wells, platforms and templates. This area represents the physical presence of cuttings rather than the extent of biological impacts.

A further component of oil and gas industry activities is the drilling of exploration, development and appraisal wells. In the period up to and including 2005 the UKDEAL and NPD datasets report a total of 1,608 of these in waters deeper than 200 m. Buffers of 21,744 m^2^ (radius ∼83 m) with overlapping boundaries merged and dissolved were also applied to these wells to estimate the spatial extent of drill cuttings. Of the wells listed, coordinates for 114 UK wells were not readily available. The buffered area for these was estimated from the mean area of the other UK wells.

### Bottom Trawling

From 1 January 2005 all vessels i) exceeding 15 m overall length operating in European waters and ii) belonging to contracting parties to the North East Atlantic Fisheries Commission (NEAFC) Vessel Monitoring System Programme over 24 m overall length operating within the NEAFC Regulatory Area (Figure 1), were required to install and operate satellite-based tracking devices [35], [36]. Vessels were required to transmit data at intervals of 2 hours or less to Fishing Monitoring Centres (FMCs) located in the States in which they were registered. (In November 2009 an amendment to the NEAFC convention required data to be transmitted at least once every hour in the NEAFC Regulatory Area [37]). Data relating to vessels operating beyond EEZs (in the NEAFC Regulatory Area) are transmitted from the flag State to NEAFC.

There was no definitive source identifying i) bottom trawling vessels, ii) where trawls started and ended and iii) the size of the gear deployed. Therefore the spatial extent of bottom trawling had to be estimated from VMS datasets. VMS data for 2005 were requested from the sources listed in Table 3. Only France, the UK and NEAFC provided data. These data comprised a reporting code, position, time, date and occasionally details of the catch. No dataset gave any indication of whether the vessel was engaged in fishing at the time the position was reported. Data supplied by the UK, covering UK waters, included information about the type of vessel (e.g. demersal trawler, purse seiner) but this was not reported for all vessels. The French dataset, covering French waters, did not include speed. This had to be calculated by reference to time and distance covered between successive reported positions.

Bottom trawling activity was inferred by examining the course of each vessel in relation to seabed contours and speed. Unlike pelagic trawlers, bottom trawlers, while fishing, are likely to follow the contours of the seafloor [38]. Additionally, deep water bottom trawlers can fish only within a limited range of speeds: 1.5–5.0 knots [3], [38] (Tables 8 and 9). The size of the fishing gear was not reported. The possible distance between trawl doors, 22 m, 80 m and 125 m was identified by reference to published literature [39] and personal communication (Dick Ferro, Fisheries Research Services, Aberdeen, UK).

**Table 8:** Spatial extent of seafloor trawled in the Hatton - Rockall area during 2005: overlapping tracks not merged.

Speeds (knots)	Area trawled based on *125 m gear width (km^2^)	Area trawled based on *80 m gear width (km^2^)	Area trawled based on **22 m gear width (km^2^)
13.0–5.0	21,346	13,631	3,738
11.5–4.5	27,487	17,619	4,855
22.0–3.0	4,255	2,711	741
31.5–5.0	37,160	23,855	6,585

Estimates based on 28 vessels engaged in bottom trawling, identified from speed profiles and pattern of activity. All overlapping tracks included in estimate.

*Dick Ferro, Fisheries Research Services, Aberdeen, personal communication.

**Hall-Spencer et al., 2002 [39].

1 Davies et al., 2007 [3].

2 ICES, 2007 [38].

3 1.5–5.0 knots encompasses the range of bottom trawling speeds referred to by Davies et al., 2007 [3] and ICES, 2007 [38].

**Table 9:** Spatial extent of seafloor trawled in the Hatton - Rockall area during 2005: overlapping tracks merged.

Speeds (knots)	Area trawled based on *125 m gear width (km^2^)	Area trawled based on *80 m gear width (km^2^)	Area trawled based on **22 m gear width (km^2^)
13.0–5.0	8,051	6,067	2,227
11.5–4.5	12,041	8,983	3,192
22.0–3.0	2,710	1,837	548
31.5–5.0	13,920	10,624	3,994

Estimates based on 28 vessels engaged in bottom trawling, identified from speed profiles and pattern of activity. Overlapping tracks merged to give single area.

* Dick Ferro, Fisheries Research Services, Aberdeen, personal communication.

** Hall-Spencer et al., 2002 [39].

1 Davies et al., 2007 [3].

2 ICES, 2007 [38].

3 1.5–5.0 knots encompasses the range of bottom trawling speeds referred to by Davies et al., 2007 [3] and ICES, 2007 [38].

The NEAFC data allowed a detailed study of just one fishery in the OSPAR area in the vicinity of Hatton and Rockall. These data were used to estimate the spatial extent of bottom trawling because it was possible to determine the relationship between vessel movements and seafloor contours. This relationship was less clear for other areas within the NEAFC Regulatory Area and within French and UK waters, consequently these areas were not included in this study.

Speed frequency profiles, produced for each vessel in the NEAFC dataset using GeoCrust2.0 software [40], were provided by ICES. These profiles identified vessels with peaks of activity the 1.5–5.0 knot range. As a further check the entire 2005 NEAFC dataset comprising 797 vessels was imported into ArcGIS and patterns of vessel activity, following seafloor contours were studied. Twenty eight vessels were identified as engaged in bottom trawling in the Hatton - Rockall area. All vessels not considered to be bottom trawling were removed from the dataset. Data for the remaining 28 vessels were filtered to remove points with speeds outside the 1.5–5.0 knots range. Data points, within the speed range but lying outside the fishing grounds, in waters too deep to bottom trawl, were also removed. Sequences of consecutive data points were considered to indicate trawling periods. It was decided that each sequence was considered to have ended when the time difference between data points exceeded 2.5 hours. This time difference was chosen because occasionally the time between consecutive signals was greater than 2 hours. The resulting dataset encompassed the full range of speeds identified for bottom trawling (1.5–5.0 knots). Three further datasets were produced for the speed ranges: 3.5–5.0 knots [3], 1.5–4.5 knots [3] and 2.0–3.0 knots [38]. Each spreadsheet was imported into ArcGIS and a point to polyline conversion used to map vessel tracks.

A limitation of this method is that although vessel activity relates to seafloor contours and speeds fall within the range of bottom trawling speeds, is it not certain when fishing gear is in contact with the seafloor. Further limitations are i) the two-hourly signal frequency gives a limited indication of the true speed and activity of vessels, ii) the distances between data points are represented by straight lines so represent the minimum distance covered, iii) the absence of information about gear type and size makes further assumptions necessary.

The estimates of spatial extent of bottom trawling represent a proportion of the true extent of this activity in the OSPAR area as they are based on an analysis of vessels operating only within the Hatton - Rockall area from the NEAFC dataset. Deep water bottom trawling also takes place on the Reykjanes Ridge, the Mid-Atlantic Ridge and the continental slope [41] but these areas were not included in this study.

## Results

### Marine Scientific Research

There was no single source for marine scientific research cruise data. The quality of station lists and cruise reports ranged from purely narrative, lacking description of equipment and latitude and longitude of sampling sites, to comprehensive, including station number, cast number, type of gear, event, date and time, decimal latitude and longitude, depth, remarks, core length where applicable and institute responsible for sample.

Table 4 shows that approximately 22 km^2^ of marine research comprised activities carried out by fisheries research vessels and approximately 4 km^2^ were attributable to non-fisheries marine research. This includes the tracks of trawls, dredges and sleds and the ‘footprint’ of individual pieces of static equipment on the seafloor such as corers and grabs, which are removed immediately and the anchor weights of moorings (∼1 m^2^) which remain on the seafloor.

The cruises mapped in this study were estimated to represent approximately 45% of all scientific cruises reported on the ROSCOP website which carried out sampling on the seafloor during 2005 in water depths greater than 200 m in the OSPAR area. Table 5 shows figures extrapolated to include the cruises for which no data were available. Extrapolating these figures gives a total spatial extent of approximately 49 km^2^ and 9 km^2^ respectively for fisheries and non-fisheries research.

For those data that were available confidence ratings of 2 and 3 denote that the location of activities were, in most instances, available but the extent of individual activities (e.g. size of equipment deployed, length of trawls) were occasionally unreported.

### Submarine Communication Cables

The data for this activity were from the two sources listed in Table 1. However, these data do not include all cables present on the seafloor. The complete dataset is only available commercially.

The results for the 4 scenarios (Table 4) considered for submarine communication cables demonstrate that this activity covers a relatively small spatial extent in all cases. The first scenario, giving an estimated 2 km^2^, represents the spatial extent of the physical presence of submarine cables for the study area. The second scenario, giving an estimated area of 4 km^2^, is independent of cable type and burial and uses a single value for cable width. The third scenario, giving an estimated area of 15 km^2^ introduces the concept of plough burial and is based on the most conservative estimate of the width of the disturbance strip, 2 m, reported in [23]. The fourth scenario, giving an estimated area of 61 km^2^, is based on the maximum estimated width of disturbance strip of 8 m [23].

The values for scenarios 1 and 2, representing an estimated 41% of all submarine communications cables, can be extrapolated to give an estimate of the total extent of this activity because they represent the physical presence of cables on or in the seabed (Table 5). The extrapolated values are 5 km^2^ and 10 km^2^ respectively. It is not appropriate to extrapolate scenarios 3 and 4 because plough burial was not introduced until the 1980s, all cables laid before that date were laid on the seabed surface.

The confidence rating of 1 and 2 denotes that while data relating to the location of submarine cables for areas to ∼25°W were available there was no specific indication of the cable diameter or whether it was buried. There was no freely available information for areas beyond 25°W.

### Waste Disposal

#### Radioactive waste

Information relating to dumping sites for radioactive waste was obtained from a single source [26], (Table 1). While the total area designated for dumping of radioactive waste was estimated to be 26,323 km^2^, based on the aggregated areas with overlapping boundaries dissolved for each of the four designated sites (Table 6) this does not represent the area of seafloor covered by drums of waste. A second estimate of ∼0.2 km^2^ was calculated based on the tonnage, estimated number of drums (Table 6) and the area of each.

The confidence rating of 2 relating to the spatial extent of this activity denotes that while the location is reported the spatial extent is based on an estimated number of drums and drum size.

#### Munitions and chemical weapons

Inadequate documentation at the time of dumping of chemical weapons and munitions and the subsequent loss or destruction of documentation means that the full extent of this activity is unknown [28]. Accurate information on the quantities, present condition and current location of these materials is lacking [5], [28], [42]. While the location and type of some conventional and chemical munitions are known, other material is reported to have been dumped outside official dumping areas [43]. Furthermore, movement across the seabed or burial through natural processes or anthropogenic activity, have complicated establishing the locations of dumped munitions [43]. The disposal of redundant munitions has continued intermittently [4]. The most recent known event occurred during 1994 when Portugal, under Sovereign Immunity, scuttled a redundant vessel loaded with >2000 t of surplus munitions 346 km from the Portuguese coast at the edge of their EEZ in >4000 m of water [44].

The total spatial extent for this activity was estimated to be 1.4 km^2^.

While information relating to munitions dumpsites was available openly online [28], lack of knowledge about the precise current location and extent of dumped material is reflected in a confidence rating of 1.

### Oil and Gas Industry

The datasets and GIS shapefiles for this activity were downloaded free of charge in February 2008. However UKDEAL shapefiles are now available only on payment of a subscription. Norwegian data remain available without charge.

The estimated spatial extent of oil and gas industry pipelines in water deeper than 200 m was 4 km^2^, while the footprint for structures on the seafloor (platforms, templates and wellheads) totalled 0.2 km^2^. This figure is likely to be an underestimate as it includes only templates, wellheads and platforms. Other equipment and activities such as anchors and rock dumps were not included. The addition of the associated cuttings piles to the latter estimate resulted in a total estimated spatial extent of 3 km^2^. The estimated spatial extent of exploration, development and appraisal wells drilled between 1960 and December 2005 together with the associated cuttings piles totalled approximately 15 km^2^ while that for the single year, 2005, totalled 1 km^2^. The total spatial extent of pipelines, structures and associated cuttings piles together with all exploration, appraisal and development wells drilled between 1960 and December 2005 and their associated cuttings piles in water deeper than 200 m was 23.2 km^2^.

Oil and gas industry installations are complex. A wide variety of equipment is used each with its own type of disturbance (e.g. rock dumps, anchors). It has not been possible to evaluate these impacts in this study because data are not readily available. Confidence ratings of 2 and 3 reflect the variations in the quality of data. The UKDEAL dataset reported both location and diameter of pipelines resulting in a confidence rating of 3. Although diameters of Norwegian pipelines were not recorded in the NPD dataset this information was available by searching for each pipeline individually in NPD Facts [31] also giving a confidence rating of 3. Neither dataset indicated the size of individual installations on the seafloor, although the location of each is reported, giving a confidence rating of 2. Similarly, the location of development, appraisal and exploration wells are reported but no indication of the extent of these activities was recorded. It was unclear what type of installation was being referred to in the NPD dataset without following a hyperlink for each individual facility. Although a description of the individual installations was given in the UKDEAL dataset (e.g. clump weight, pipe crossing, wellhead) no indication of dimensions was included.

### Bottom Trawling

As there was no definitive source identifying i) bottom trawling vessels, ii) where trawls started and ended and iii) the size of the gear deployed the spatial extent of bottom trawling had to be estimated from analysis of VMS datasets. Willingness to provide VMS datasets varied between States. Only two States out of the nine to which requests for data were made provided VMS datasets.

Table 8 shows the total area of seafloor trawled for each speed range, calculated by applying buffering to the vessel tracks of 22 m [39] , 80 m and 125 m, the possible spreads of the trawl doors. The least possible area trawled, 741 km^2^, relates to the narrowest speed range of 2.0–3.0 knots and gear width of 22 m (Tables 4 and 8). The greatest possible area trawled, 37,160 km^2^ relates to the widest speed range of 1.5–5.0 knots and gear width of 125 m (Tables 4 and 8).

Table 9 shows the spatial extent of bottom trawling when overlapping tracks were merged. Even if multiple trawls pass over a section of seafloor during the year only a single area is recorded. The least possible area trawled, 548 km^2^, relates to the narrowest speed range of 2.0–3.0 knots and gear width of 22 m (Tables 4 and 9). The greatest possible area trawled, 13,920 km^2^ relates to the widest speed range of 1.5–5.0 knots and gear width of 125 m (Tables 4 and 9).

**Figure 3:**
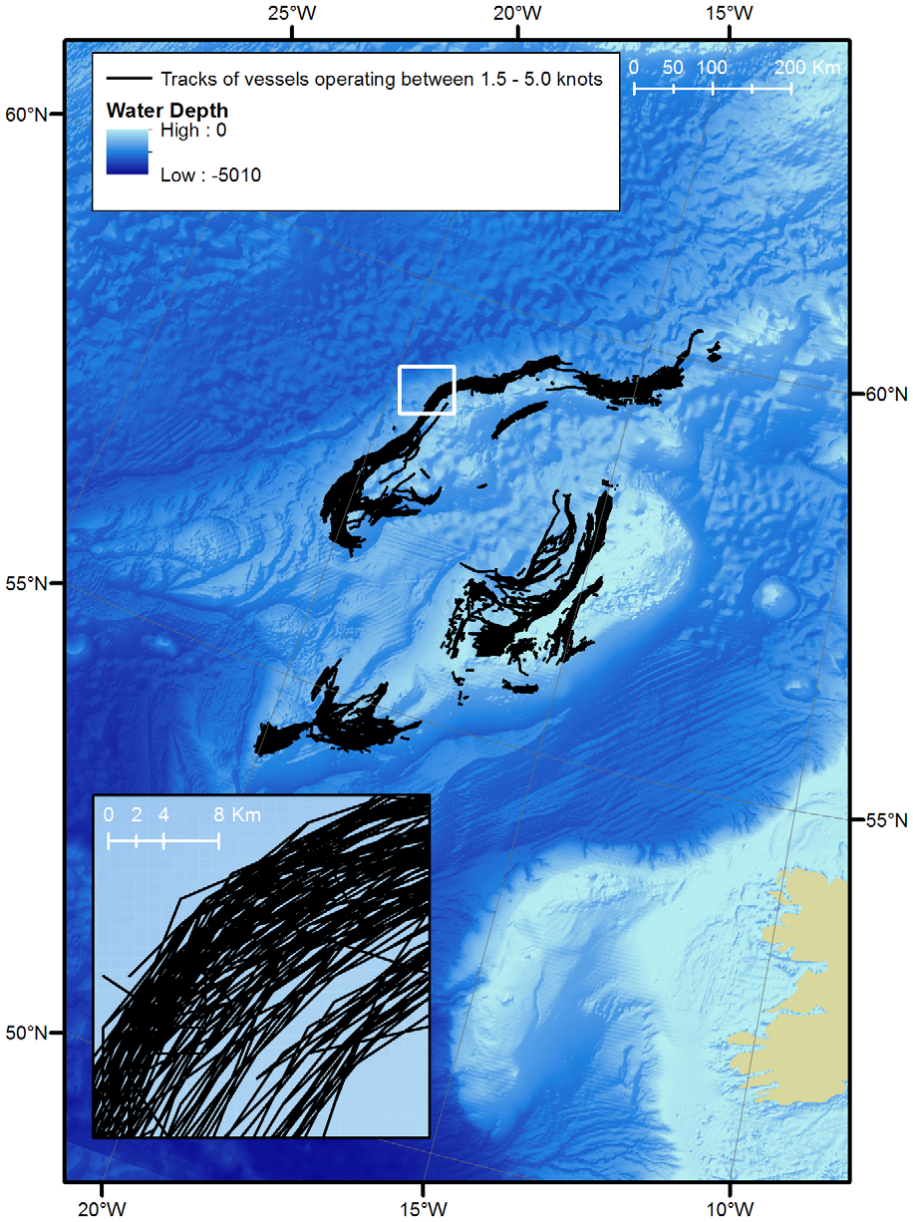
Bottom trawling. Tracks of vessels operating between 1.5 and 5.0 knots in the Hatton - Rockall area during 2005.

Figure 3 shows the distribution of this activity in the Hatton - Rockall area.

The spatial extent of bottom trawling during 2005 in the Hatton - Rockall area is greater than that of any other activity in the OSPAR region. The most conservative estimate of 548 km^2^ is one order of magnitude greater than the largest estimate for impacts by the oil and gas industry, while the estimate of 13,920 km^2^, based on the widest gear (125 m) and widest speed range (1.5–5.0 knots) with overlapping tracks merged is three orders of magnitude greater. The spatial extent for the two scenarios above without merging overlapping tracks is 741 km^2^ and 37,160 km^2^ respectively. This suggests that much of the seafloor was trawled more than once during the year.

Calculations for the spatial extent of bottom trawling were based on data from only one part of the OSPAR area - Hatton - Rockall. Extrapolations have been made based on the estimate that the Hatton - Rockall area comprises ∼50% of the deep sea trawling grounds in the OSPAR area (Table 5). The estimate for the most conservative speed range and gear width (2.0–3.0 knots, 22 m) with overlapping tracks not merged is an extrapolated value of 1,482 km^2^. The widest speed range and gear width (1.5–5.0 knots, 125 m) with overlapping tracks not merged gives an extrapolated value of 74,320 km^2^.

The extrapolated estimate for the most conservative speed range and gear width (2.0–3.0 knots, 22 m) with overlapping tracks merged is an extrapolated value of 1,096 km^2^. The widest speed range and gear width (1.5–5.0 knots, 125 m) with overlapping tracks merged gives an extrapolated value of 27,840 km^2^.

The confidence rating of 1–2 (Table 4) reflects that while VMS data indicate the position of vessels and fishing can be inferred from speed and course, neither the location nor extent of the bottom impact i.e. actual trawling were reported.

## Discussion

The results in Tables 4 and 5 are a first attempt to quantify the extent of human activities in the deep North East Atlantic together with an evaluation of confidence in the data. It is not practicable to present a definitive, unequivocal value for each activity as each encompasses a range of alternatives. Variables include the size of fishing gear, speed ranges within which vessels can operate, width of submarine cables, buried or non-buried cables, the size of individual oil and gas industry installations and extent of cuttings piles. Nevertheless, the figures presented represent the best estimates available and we have provided estimates based on both high and low extremes e.g. for the fishing data. This study has highlighted how complex it is to determine impacts in the deep-sea and how difficult it is to establish a comprehensive baseline for management.

Although the principal scope of this study is to establish the spatial extent of each activity it is worth noting that while some activities have an immediate impact after which seafloor communities may be re-established (albeit on perhaps long timescales), other activities, such as waste disposal, may have an effect for many years and the impact is likely to extend far beyond the physical disturbance.

The results demonstrate that the extent of human activities on the deep-sea floor in the OSPAR area of the North East Atlantic varies widely. Of the activities assessed dumping of waste was found to have the lowest spatial extent. The combined total of radioactive waste, munitions and chemical weapons dumpsites was found to be 1.6 km^2^. The strategy of sea disposal of low level radioactive waste was one of dispersal and dilution rather than containment [45]. The lifetime of the iron drums containing the waste was estimated to be between 15–150 years while bitumen or concrete blocks encasing waste were estimated to last 1000 years [26]. So, although the dumping has ceased, such material may still leak from containers into the environment [26]. The main source of artificial radionuclides in the deep North East Atlantic is from atomic weapons testing carried out during 1960s. However, ^233^Pu/^239+240^Pu isotopic ratios in some samples of the fish *Coryphaenoides armatus* suggest an influence from the dumped material [46]. Similarly, while the spatial extent of munitions and chemical weapons dumpsites, estimated to be 1.4 km^2^, is a relatively small area, the presence of this material poses a significant risk, particularly when disturbed [28].

Non-fisheries marine scientific research has a relatively small footprint. It is usually carried out by academic institutions using a range of equipment on the seafloor to sample the marine environment including moorings, grabs, corers, dredges and trawls. Much of this equipment has only a single impact of a few square meters. Considerably more research is carried out by academic institutions or fisheries research laboratories to determine fish population size and distribution. The spatial extent of fisheries marine scientific research is moderate. While fisheries research also involves the deployment of sampling equipment, such as grabs and moorings, it involves a higher proportion of bottom impact trawling.

The spatial extent of telecommunication cables is low to moderate depending on the whether cable burial is included in the calculation. The maximum extent of this activity (61 km^2^), based on an 8 m wide disturbance strip in water depths between 200–1,500 m is likely to be an overestimate. This is because about 20% of cables in 200–1,500 m water depth are not buried and an 8 m wide disturbance strip may be an overestimate in many cases.

The spatial extent of oil and gas industry activities is moderate. While structures such as templates, wellheads, platforms and cuttings piles have been included in the estimates it is likely that this is an underestimate as other equipment and activities, for example, weights, anchors, rock dumps are not included.

A major finding of this study is that the spatial extent of bottom trawling is orders of magnitude greater than that for the other activities assessed. Even on the lowest possible estimates it is an order of magnitude greater than the sum of all the other activities. Despite the extent of this activity the total global catch from bottom fisheries - longliners, gillnetters and bottom trawlers - contributed only 0.31% to the total marine capture during 2006 [47].

The maximum total area impacted by the various activities discussed here is 27,932 km^2^ (Table 5, based on the merged trawler tracks and 50 mm cable diameter data). This is a very small percentage of the total OSPAR area (11,032,175 km^2^), but such a calculation does not provide useful information. An analogy would be the area of annual destruction of Amazon rainforest as a percentage of the landmass of South America, which would mean far less than destruction as a percentage of the total area of the rainforest. Human activities are concentrated in certain areas and particularly in shallower depths. The OSPAR area also comprises many different habitats each with different and diverse ecosystems. The percentage impact in each of these habitats would provide important information but unfortunately there is virtually no detailed seabed mapping to provide this information.

### Conclusions

To meet future ecosystem-based management and governance objectives for the deep sea significant improvements are required in data collection and availability as well as a greater awareness of the relative impact of each human activity. In this paper we have shown the relative physical impacts of different activities with non-fisheries scientific research, submarine communication cables and waste disposal having low physical impacts whilst oil and gas activities and fisheries scientific research have moderate impacts. The impact of bottom trawling is at least an order of magnitude greater than all the other activities combined.
